# *Rhodococcus* Infection in Solid Organ and Hematopoietic Stem Cell Transplant Recipients^1^

**DOI:** 10.3201/eid2303.160633

**Published:** 2017-03

**Authors:** Pascalis Vergidis, Ella J. Ariza-Heredia, Anoma Nellore, Camille N. Kotton, Daniel R. Kaul, Michele I. Morris, Theodoros Kelesidis, Harshal Shah, Seo Young Park, M. Hong Nguyen, Raymund R. Razonable

**Affiliations:** University of Pittsburgh School of Medicine, Pittsburgh, Pennsylvania, USA (P. Vergidis, S.Y. Park, M.H. Nguyen);; University of Texas MD Anderson Cancer Center, Houston, Texas, USA (E.J. Ariza-Heredia);; University of Alabama, Birmingham, Alabama, USA (A. Nellore);; Massachusetts General Hospital, Boston, Massachusetts, USA (C.N. Kotton);; University of Michigan Medical School, Ann Arbor, Michigan, USA (D.R. Kaul);; University of Miami Miller School of Medicine, Miami, Florida, USA (M.I. Morris);; University of California David Geffen School of Medicine, Los Angeles, California, USA (T. Kelesidis);; Mayo Clinic, Jacksonville, Florida, USA (H. Shah);; Mayo Clinic, Rochester, Minnesota, USA (R.R. Razonable)

**Keywords:** Rhodococcus, bacteria, infection, solid organ transplant, hematopoietic stem cell transplant, transplant recipients, opportunistic infection

## Abstract

We conducted a case–control study of 18 US transplant recipients with *Rhodococcus* infection and 36 matched controls. The predominant types of infection were pneumonia and bacteremia. Diabetes mellitus and recent opportunistic infection were independently associated with disease. Outcomes were generally favorable except for 1 relapse and 1 death.

*Rhodococcus*, a gram-positive coccobacillus, has been isolated from water, soil, and the manure of herbivores. It is a facultative intracellular pathogen that survives in host macrophages. Immunosuppressive medications that compromise cell-mediated immunity can predispose to infection (*1*,*2*).

Our knowledge of disease characteristics among transplant recipients is limited to case reports (*3*–*8*). With the increase in organ transplantation and improved survival of transplant recipients, the incidence of disease will likely increase in the coming years. In this study, we sought to describe characteristics, risk factors, and outcomes of *Rhodococcus* infection among solid organ transplant (SOT) and hematopoietic stem cell transplant (HSCT) recipients in the United States.

## The Study

We conducted a case–control study at 8 US medical centers during January 2000–December 2012. The study was approved by appropriate Institutional Review Boards. Case-patients were those with clinical or radiographic features of infection and positive culture results for *Rhodococcus* spp. Identification of the organism was performed by using biochemical methods at microbiology laboratories of participating institutions. At the discretion of laboratory staff, identification was confirmed by using 16S rRNA sequencing.

Controls received a similar organ within 3 months before or after the index case-patient at the same center and did not show *Rhodococcus* infection. Each case-patient was matched with 2 controls. Allogeneic and autologous HSCT recipients were matched to recipients of the same type.

We used descriptive statistics to summarize results for the cohort. Conditional logistic regression was used to evaluate risk factors for infection. Factors associated with disease less than the 0.10 significance level for univariate analysis were included in the multivariate model. Statistical software Stata/SE version 13.1 (StataCorp LP, College Station, TX, USA) was used.

We identified 18 patients with *Rhodococcus* spp. infection (Table 1). Mean age was 55 (range 3–78) years. Six patients underwent HSCT (5 allogeneic, 1 autologous) and 12 SOT (4 heart, 4 lung, 3 kidney, 1 liver). Median time from transplant to infection was 5 (range 2–54) for HSCT recipients and 28 (range 3–237) months for SOT recipients. Infection occurred within the first year posttransplant for half of the patients. Five (39%) of 13 patients were living on a farm or had known contact with horses; exposure history was unknown for 5 patients.

**Table 1:** Clinical characteristics, treatment, and outcome for 18 patients infected with *Rhodococcus* spp, United States*

Patient age, y/sex	Residence	Transplant, underlying disease	Time from transplant to infection	Clinical feature	Lung pathology	Immunosuppression at infection	Antimicrobial drug treatment	Treatment duration	Outcome at 90 d†
76/M	Wisconsin	Kidney, obstructive uropathy	4 y	Pneumonia (productive cough, chest pain)	Chronic inflammation and reactive changes	Tacrolimus, prednisone	Ertapenem, levofloxacin → moxifloxacin	12 mo	Partial response
57/F	Iowa	Heart, ischemic cardiomyopathy	5 mo	Catheter-associated bacteremia	NT	Sirolimus, MMF, prednisone	Vancomycin, levofloxacin → levofloxacin	7 mo	Complete response
76/M	Minnesota	Heart, ischemic cardiomyopathy	20 y	Pacemaker pocket wound infection	NT	Cyclosporine, MMF, prednisone	TMP/SMX	2 mo	Complete response
59/F	Massachusetts	Double lung, usual interstitial pneumonia	8 mo	Bacteremia	NT	Cyclosporine, prednisone	Vancomycin → azithromycin	60 mo	Complete response
45/M	Massachusetts	Double lung, lymphangiolyomatosis	5 y	Pneumonia (fever, dyspnea), bacteremia	NT	Tacrolimus, MMF, prednisone	Vancomycin, meropenem → azithromycin, rifampin	8 mo	Unknown response
78/F	Massachusetts	Kidney, diabetic nephropathy	7 y	Pneumonia (productive cough)	Small chronic granulomas admixed with reactive bronchial cells	Tacrolimus, MMF, prednisone	Moxifloxacin → doxycycline	14 mo	Stable disease
53/M	Michigan	Heart, ischemic cardiomyopathy	10 mo	Pneumonia (cough), bacteremia	NT	Tacrolimus, MMF, prednisone	Vancomycin → levofloxacin, azithromycin	5 mo	Partial response
54/M	Michigan	Kidney, glomerulopathy	4 mo	Pneumonia (cough)	NT	Tacrolimus, MMF, prednisone	Meropenem, azithromycin, meropenem	3 mo	Partial response
68/M	Florida	Heart, ischemic cardiomyopathy	3 mo	Pneumonia (fever)	NT	Tacrolimus, MMF	Vancomycin, imipenem, TMP/SMX → imipenem, ciprofloxacin	11 mo	Partial response
53/M	Florida	Liver, alcoholic cirrhosis	34 mo	Catheter-associated bacteremia	NT	Sirolimus, MMF	Vancomycin, ceftriaxone	3 mo	Complete response
67/F	Pennsylvania	Left lung, idiopathic pulmonary fibrosis	5 y	Pneumonia (cough, dyspnea)	NT	Tacrolimus, MMF, prednisone	Vancomycin, azithromycin	2 mo	Complete response
51/M	Nebraska	Double lung, scleroderma with pulmonary fibrosis	22 mo	Pneumonia (fever, cough, dyspnea, chest pain)	NT	Tacrolimus, MMF, prednisone	Minocycline, azithromycin → azithromycin, linezolid	6 mo	Partial response
14/M	California	Allogeneic HSCT, acute myeloid leukemia	6 mo	Catheter-associated bacteremia	NT	Tacrolimus, prednisone	Linezolid	2 wk	Complete response
61/M	Mississippi	Allogeneic HSCT, chronic lymphocytic leukemia	54 mo	Pneumonia (fever, dyspnea), bacteremia	Acute pulmonary inflammation	Prednisone	Meropenem, tigecycline → ertapenem, TMP/SMX, tigecycline	6 mo	Stable response, disease relapse at 9 mo
65/F	Texas	Allogeneic HSCT, follicular lymphoma	8 mo	Pneumonia	Focal tissue, eosinophilia, and Masson bodies; organizing pneumonia	None	Gatifloxacin, azithromycin	2 mo	Complete response
3/M	Texas	Autologous HSCT, neuroblastoma	2 mo	Bacteremia	NT	None	Vancomycin, gatifloxacin	1 mo	Complete response
63/M	Texas	Allogeneic HSCT, acute myeloid leukemia	4 mo	Catheter-associated bacteremia	NT	Tacrolimus, prednisone for GVHD	Vancomycin	1 mo	Complete response
53/F	Texas	Allogeneic HSCT, chronic myelogenous leukemia	4 mo	Pneumonia (fever, dyspnea, productive cough, chest pain), bacteremia	NT	Sirolimus for GVHD	Tigecycline, TMP/SMX, piperacillin/tazobactam → levofloxacin, TMP/SMX, piperacillin/tazobactam	2 wk	Died of respiratory failure/ARDS at day 13

*ARDS, acute respiratory distress syndrome; GVHD, graft-versus-host disease; MMF, mycophenolate mofetil; NT, not tested; TMP/SMX, trimethoprim/sulfamethoxazole; →, change in treatment regimen. †Outcome (response to treatment) was categorized as complete (resolution of all attributable signs, symptoms, and radiographic abnormalities); partial (clinical improvement and >50% improvement in radiographic abnormalities); stable (no improvement in clinical manifestations and <50% improvement in radiographic findings); or failure (deterioration of condition or death).

Median time to diagnosis after onset of symptoms was 20 (range 2–67) days. This median was determined mainly by the time that the patient sought medical attention and the time of clinical specimen collection. At the time of diagnosis, 3 (17%) patients were managed in outpatient settings, 12 (67%) in inpatient wards, and 3 (17%) in intensive care units.

The predominant infections were pneumonia (61%, 11/18) and bacteremia (56%, 10/18). Bacteremia was secondary to pneumonia for 4 patients and catheter-associated for 4 patients. Fever occurred in half of the patients. Patients with pneumonia had dyspnea (45%, 5/11), cough (70%, 7/10), sputum production (20%, 2/10), and chest pain (30%, 3/10). None had hemoptysis. Lung disease was infiltrative in 8/11 (73%) patients, nodular in 8/11 (73%), and cavitary in 2/11 (18%). Median neutrophil count at diagnosis was 4,133/mm^3^ (range 532–16,468/mm^3^), and median lymphocyte count was 705/mm^3^ (range 90–3,350/mm^3^). Species identification was performed for 9 isolates (8 *R. equi* and 1 *R. corynebacterioides*). We found no significant difference in incidence of preceding opportunistic infection between SOT and HSCT recipients (33% vs. 50%; p = 0.62). 

Infected patients were matched with 36 controls. Univariate analysis showed that type of immunosuppression, augmented immunosuppression, increased levels of tacrolimus or cyclosporine, and trimethoprim/sulfamethoxazole (TMP/SMX) prophylaxis were not associated with infection. Multivariate analysis showed that diabetes mellitus (p = 0.041) and recent opportunistic infection (p = 0.045) were independently associated with infection (Table 2).

**Table 2:** Univariate analysis of risk factors associated with *Rhodococcus* infection in solid organ and hematopoietic stem cell transplant recipients, United States*

Variable	Case-patients, n = 18	Control patients, n = 36	Univariate OR (95% CI)	p value
Mean age, y (range)	55 (3–78)	50 (2–78)	1.05 (0.99–1.11)	0.13
Male sex	12/18 (66.7)	22/36 (61.1)	1.21 (0.42–3.45)	0.72
White race	15/18 (83.3)	23/36 (63.9)	3.17 (0.65–15.43)	0.15
Diabetes mellitus†	9/18 (50.0)	6/34 (17.6)	9.90 (1.20–81.62)	0.03
Chronic kidney disease‡	3/16 (18.8)	5/35 (14.3)	1.15 (0.19–7.03)	0.88
Immunosuppressant				
Tacrolimus	10/18 (55.6)	25/35 (71.4)	0.15 (0.02–1.39)	0.10
Sirolimus	3/18 (16.7)	2/35 (5.7)	4.65 (0.46–46.89)	0.19
Mycophenolate mofetil	10/18 (55.6)	18/35 (5.4)	1.36 (0.20–9.0)	0.75
Prednisone	13/18 (72.2)	25/35 (71.4)	1.00 (0.25–4.0)	1.00
Cyclosporine	2/18 (11.1)	3/35 (8.6)	2.00 (0.13–31.98)	0.62
Increased calcineurin inhibitor level§	2/13 (15.4)	4/32 (13.3)	1.20 (0.16–9.20)	0.86
History of allograft rejection	2/12 (16.7)	1/24 (4.2)	4.00 (0.36–44.11)	0.26
Augmented immunosuppression¶	7/18 (38.9)	13/37 (35.1)	1.28 (0.24–6.89)	0.77
TMP/SMX prophylaxis	10/18 (55.6)	19/36 (52.7)	1.15 (0.33–4.03)	0.83
History of opportunistic infection#	7/18 (38.9)	4/36 (11.1)	10.57 (1.25–89.0)	0.03

*Values are no. (%) unless otherwise indicated. OR, odds ratio; TMP/SMX, trimethoprim/sulfamethoxazole. †Requiring treatment with oral antidiabetic agent(s) or insulin. ‡Creatinine level >2 mg/dL. §Tacrolimus >12 μg/mL or cyclosporine >250 μg/mL in the preceding 30 days. ¶Use of corticosteroid pulses, alemtuzumab, anti-thymocyte globulin, basiliximab, or rituximab in the 6 months preceding infection. #Opportunistic infections in case-patients were cytomegalovirus viremia (3) or invasive disease (2), pulmonary aspergillosis (1), and BK polyomavirus–associated hemorrhagic cystitis (1).

All isolates tested were susceptible to vancomycin (5/5), rifampin (5/5), linezolid (9/9), and imipenem (7/7). Fourteen percent (1/7) were susceptible to amoxicillin/clavulanate; 29% (2/7) to ceftriaxone; 55% (6/11) to TMP/SMX; 70% (7/10) to tetracycline or minocycline; 75% (6/8) to azithromycin or clarithromycin; and 80% (4/5) to levofloxacin, moxifloxacin, or gatifloxacin. Four isolates were resistant to penicillin and 1 was resistant to clindamycin.

Most patients received combination treatment with 2–3 antimicrobial drugs (Table 1). Most commonly used drugs in the initial regimen were vancomycin, a fluoroquinolone, or a carbapenem. Median duration of treatment was 1 month (range 2 weeks–7 months) for patients with cathether-associated bacteremia and 6 months (range 2–60 months) for patients with all other infections. Immunosuppression was decreased in 44% (7/16). The patient with a pacemaker pocket infection had the device removed.

Median follow-up period was 17 (range 1–84) months. One allogeneic HSCT recipient with *R. equi* pneumonia died of respiratory failure 13 days after diagnosis. He was receiving effective treatment with levofloxacin and TMP/SMX. One allogeneic HSCT recipient with bacteremic cavitary pneumonia who received 6 months of antimicrobial drug treatment had disease relapse (fever, cough, and dyspnea) 9 months after initial presentation. *Rhodococcus* spp. were recovered from bronchoalveolar lavage fluid at relapse. The 4 patients with catheter-associated bacteremia had their catheters removed and did not show relapse.

## Conclusions

Our study showed an association between *Rhodococcus* infection and preceding opportunistic infection. This finding suggests that affected patients have a high net state of immunosuppression. Prior cytomegalovirus infection, the most common opportunistic infection in the study, might have had an immunomodulatory effect that made patients more likely to show development of a second opportunistic infection.

Most patients were not neutropenic at diagnosis, consistent with the fact that *Rhodococcus* spp. affect mainly patients with impaired cell-mediated immunity. TMP/SMX prophylaxis did not confer protection, probably because of high resistance rates. Patients did not always have a history of exposure to livestock as previously described (*2*). Median time to infection was shorter for HSCT recipients, probably because catheter-associated bacteremia was more common among these patients. Predominantly among SOT recipients, infection occurred late after transplant (>12 months), and none of the infections were catheter-associated (*2*).

For systemic infections, monotherapy might result in emergence of resistance. In a report from Taiwan, 3 of 7 *R. equi* isolates had inherent concomitant resistance to all β-lactams, macrolides, and rifampin (*9*). We did not observe this multidrug-resistance pattern. For transplant recipients with systemic infection, we recommend combination treatment with 2–3 antimicrobial drugs (vancomycin, fluoroquinolone, or carbapenem). TMP/SMX and clindamycin should be avoided in empiric treatment regimens because of variable rates of susceptibility. Macrolide antimicrobial drugs, except for azithromycin, decrease the metabolism of cyclosporine, tacrolimus, sirolimus, and everolimus. Conversely, rifampin increases the metabolism of these drugs. These interactions should be considered when treating transplant recipients. *Rhodococcus* spp. can also form adherent biofilms (*10*). Thus, removal of central catheters is imperative in the management of infected patients.

We observed only 1 death attributable to infection in an HSCT recipient. This finding differs from an attributable mortality rate of 34.3% in a multicenter study of 67 patients with AIDS (mean CD4 cell count 35/μL) (*11*). The higher mortality rate probably reflects the degree of immunosuppression among patients with advanced HIV infection and close clinical monitoring of transplant recipients, which enables timely management. Death and relapse rates in our series were comparable with those in a review of 30 cases (*2*).

Our retrospective study had inherent limitations related to collection of data. In the analysis, we included SOT and HSCT recipients who differed in underlying disease states and immunosuppression. The study was also limited by the relatively small number of cases. This limitation was also reflected in wide CIs in risk factor analysis. However, we showed that risk factors for *Rhodococcus* infection were diabetes mellitus and recent opportunistic infection. Outcomes were generally favorable after appropriate and timely treatment.
